# Supplementary material for: Versatile cell-based assay for measuring DNA alkylation damage and its repair
Source: Sci Rep. 2021 Sep 15;11:18393. doi: 10.1038/s41598-021-97523-w (PMC8443546; doi:10.1038/s41598-021-97523-w)
Supplement: Supplementary file 1 — Supplementary Information. [file 41598_2021_97523_MOESM1_ESM.docx]

**Supplementary Information**

**
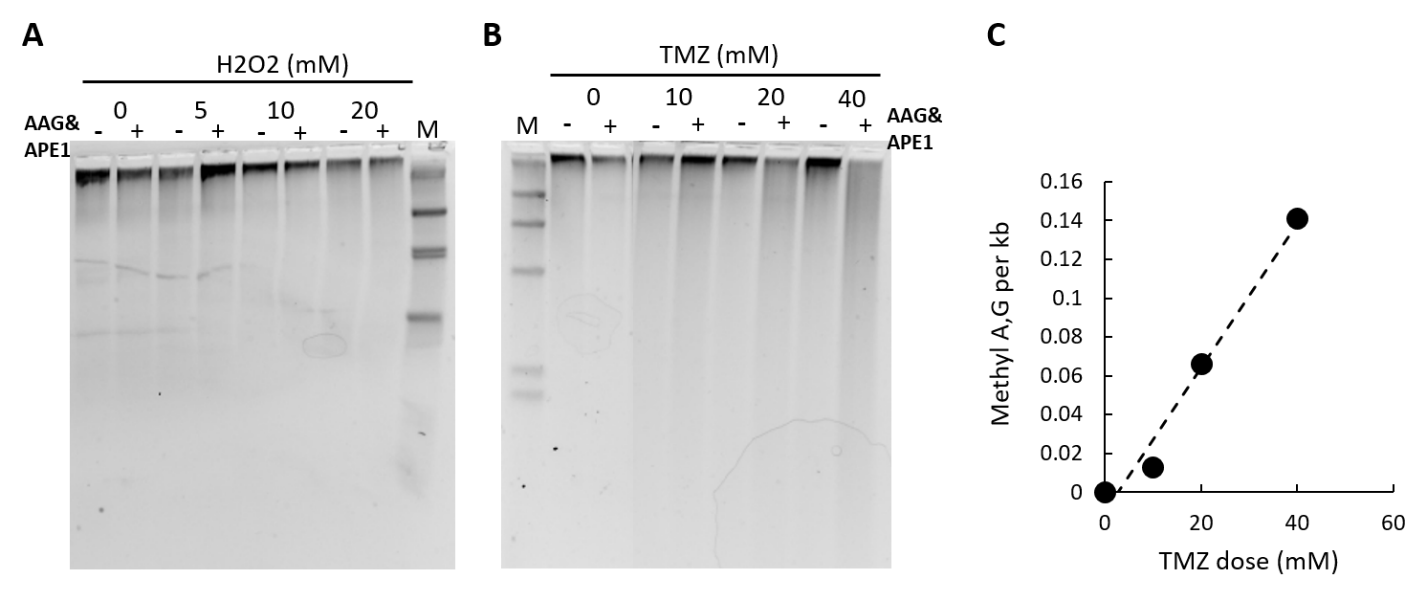
**

**Figure S1. Specificity of alk-BER.** **A)** Hydrogen peroxide (H2O2) induced DNA damage. SW13 cells were exposed to increasing doses of H2O2 for 5min at RT, followed by DNA purification, AAG&APE1 digest and alkaline agarose gel electrophoresis. **B)** Temozolomide (TMZ) induced DNA damage. SW13 cells were exposed to increasing doses of TMZ for 10 min at RT, followed by DNA purification, AAG&APE1 digest and alkaline agarose gel electrophoresis and data quantification. **C)** Quantification data of TMZ dose dependent accumulation of methyl A,G per kb DNA fragment.

**
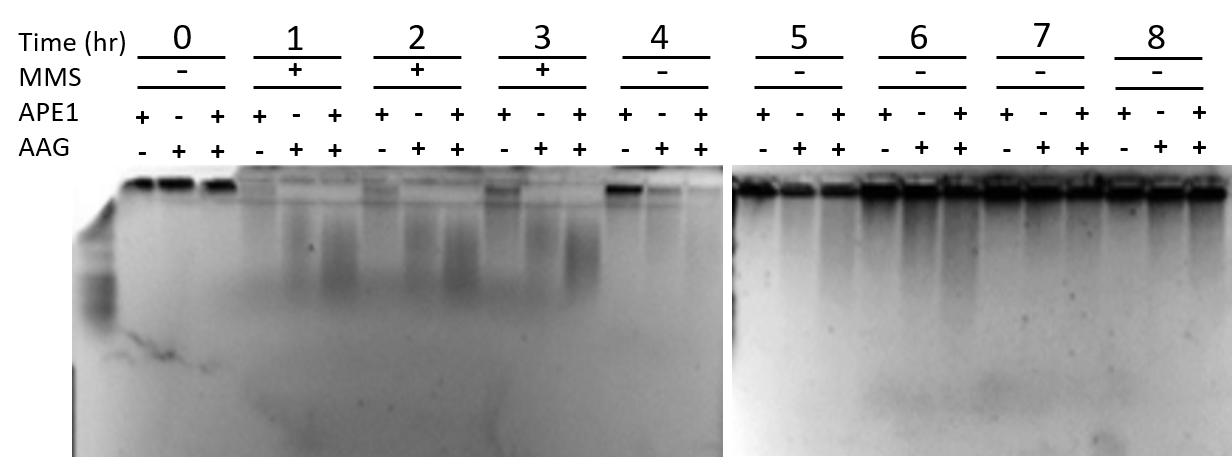
**

**Figure S2. Alk-BER assay in *Neurospora crassa*.** Representative alkaline agarose gel image of MMS-induced DNA damage, followed by DNA repair in the wild type strain of *Neurospora*, 0: control genomic DNA from cells not exposed to MMS; 1-3 hours: DNA from cells exposed to 3.5mM MMS continuously for 1, 2 and 3 hours respectively; 4-8 hours: DNA from cells that were allowed to repair DNA in media without MMS for 1-4 hours respectively. Each DNA sample was treated with combination of human APE1 and AAG enzymes: APE1 (+) and without AAG (-), without APE1 (-) and with AAG (+), and with both enzymes APE (+) & AAG (+).


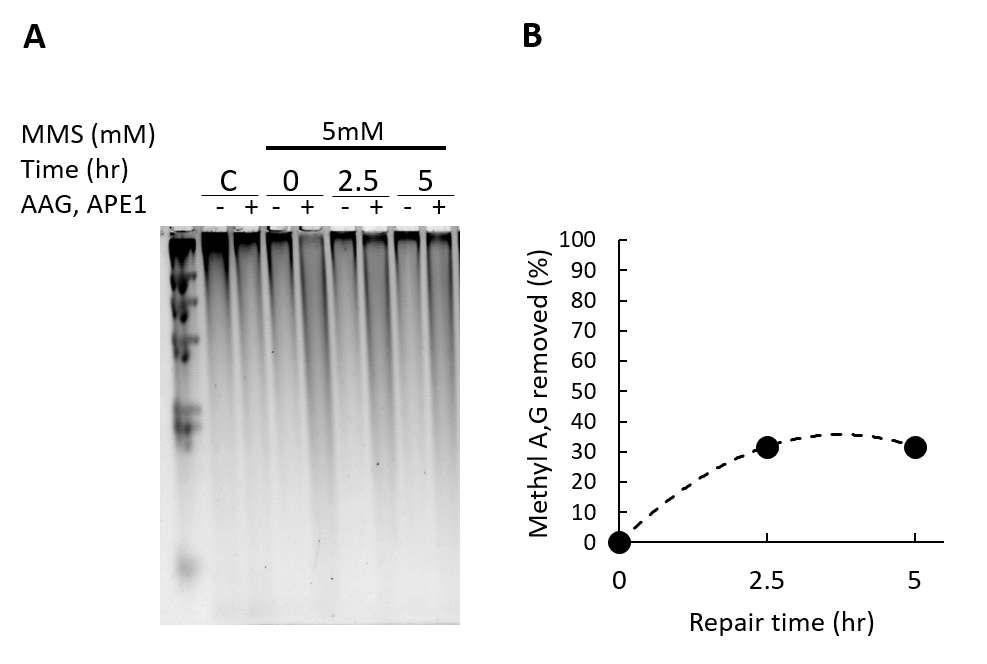


**Figure S3. Alk-BER assay in human lymphoblastoid cell line GM12878.** The representative alkaline agarose gel illustrates DNA damage and repair time course performed with GM12878 cells. Cells were treated with 5mM MMS for 5 minutes, MMS was removed and cells were allowed to repair DNA for 2.5 and 5 hours.

**Uncropped gel images:**

**Uncropped gel image, Figure 2A.**

**
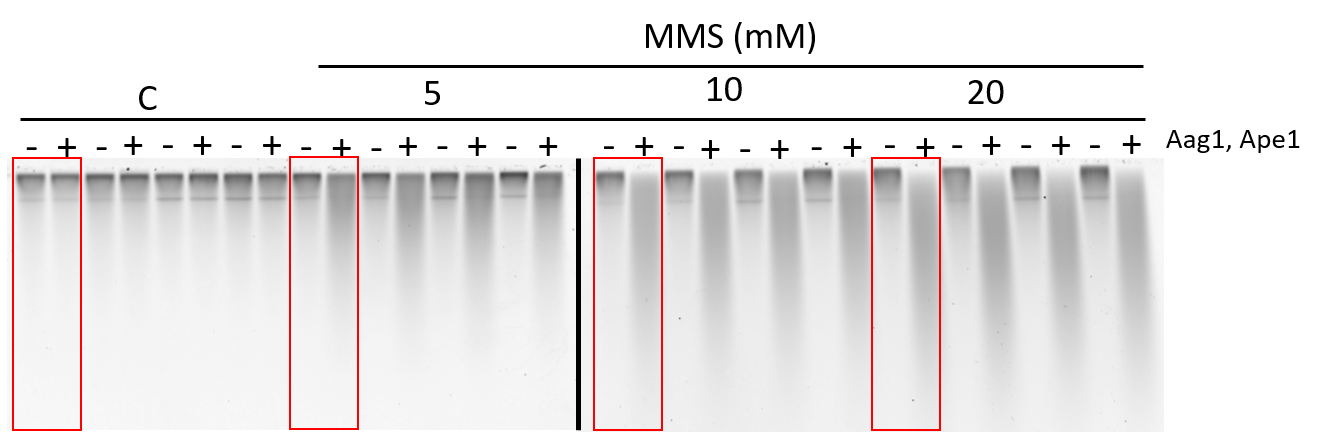
**

**Uncropped gel image, Figure 4C.**


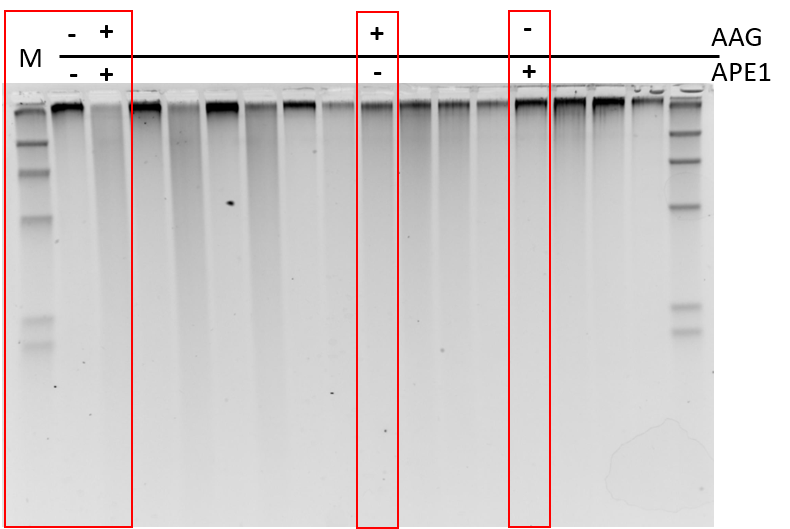


**Uncropped Western Blot image, Figure 5B.**

**
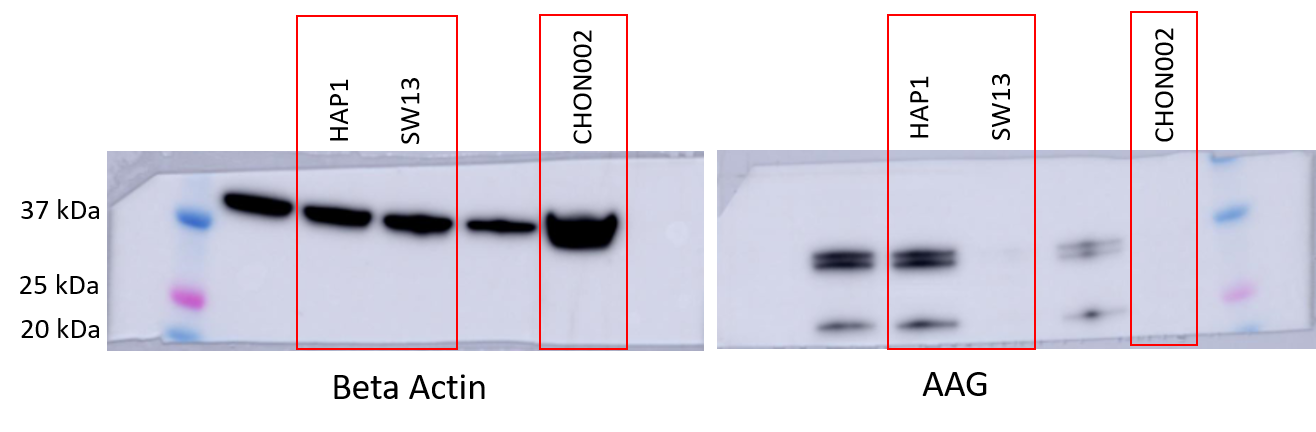
**
